# Structural behavior of laser-irradiated γ-Fe_2_O_3_ nanocrystals dispersed in porous silica matrix : γ-Fe_2_O_3_ to α-Fe_2_O_3_ phase transition and formation of ε-Fe_2_O_3_

**DOI:** 10.1080/14686996.2016.1222494

**Published:** 2016-09-29

**Authors:** Yassine El Mendili, Jean-François Bardeau, Nirina Randrianantoandro, Jean-Marc Greneche, Fabien Grasset

**Affiliations:** ^a^ Institut des Molécules et Matériaux du Mans, UMR CNRS 6283, LUNAM Université, Le Mans, France; ^b^ Institut des Sciences Chimiques de Rennes, CNRS UMR 6226, Université Rennes 1, Rennes, France; ^c^ CNRS-Saint Gobain, Laboratory for Innovative Key Materials and Structures, UMI 3629 LINK, National Institute of Material Science, Tsukuba, Japan

**Keywords:** Nanocomposites, sol-gel, laser irradiation, Raman, phase transformations, silica matrix, ε-Fe_2_O_3_, 10 Engineering and Structural materials, 103 Composites, 212 Surface and interfaces, 306 Thin film / Coatings

## Abstract

The effects of laser irradiation on γ-Fe_2_O_3_ 4 ± 1 nm diameter maghemite nanocrystals synthesized by co-precipitation and dispersed into an amorphous silica matrix by sol-gel methods have been investigated as function of iron oxide mass fraction. The structural properties of γ-Fe_2_O_3_ phase were carefully examined by X-ray diffraction and transmission electron microscopy. It has been shown that γ-Fe_2_O_3_ nanocrystals are isolated from each other and uniformly dispersed in silica matrix. The phase stability of maghemite nanocrystals was examined *in situ* under laser irradiation by Raman spectroscopy and compared with that resulting from heat treatment by X-ray diffraction. It was concluded that ε-Fe_2_O_3_ is an intermediate phase between γ-Fe_2_O_3_ and *α*-Fe_2_O_3_ and a series of distinct Raman vibrational bands were identified with the ε-Fe_2_O_3_ phase. The structural transformation of γ-Fe_2_O_3_ into *α*-Fe_2_O_3_ occurs either directly or via ε-Fe_2_O_3_, depending on the rate of nanocrystal agglomeration, the concentration of iron oxide in the nanocomposite and the properties of silica matrix. A phase diagram is established as a function of laser power density and concentration.

## Graphical abstract

**Figure:**
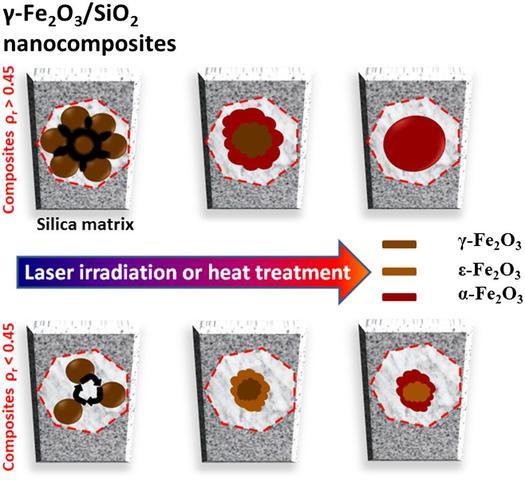

## 1. Introduction

The properties of iron oxide nanomaterials have recently attracted much interest because of their applications in active catalytic, magnetic, nonlinear optics materials and photo-electrodes.[1–3] Magnetic nanoparticles (NPs) have received considerable attention in the last decade, particularly in nanotechnology because NPs offer interesting magnetic and surface properties while their nontoxicity, biodegradability and biocompatibility allow applications in biomedicine and biotechnology.[4–9]

Iron oxide is widely used in industry. Among the four Fe_2_O_3_ polymorphs (alpha, beta, gamma, and epsilon), maghemite (γ-Fe_2_O_3_) has been selected for potential medical applications.[7–10] Maghemite is a metastable polymorph of the thermodynamically more stable hematite (*α*-Fe_2_O_3_). The temperature of transformation for the phase transition γ-Fe_2_O_3_ to *α*-Fe_2_O_3_ is estimated at 400 °C for bulk material. The transition temperature and the mechanism of the structural transformation can be largely influenced by numerous factors, such as particle size, pressure, lattice defects, surface phenomena and functionalization.[9–12] Recently, we reported how the phase transition from γ‑Fe_2_O_3_ to *α*‑Fe_2_O_3_ NPs can be induced by thermal treatment and laser irradiation.[13,14] We showed that in both cases this transformation was associated with an increase in the grain sizes induced by the coalescence and the agglomeration of nanocrystals. Attempts have already been made to stabilize the nanometric γ-Fe_2_O_3_ phase by functionalizing NPs with organic molecules [5,15] or by dispersing maghemite in a polymeric, glassy, metallic or ceramic matrix.[16–20] Among the different procedures reported in literature, the sol-gel method [21] has been proven to offer many advantages. In the case of nanocomposites consisting of maghemite nanocrystals dispersed in a silica matrix, the matrix stabilizes the nanocrystals, delaying their thermal transformation into *α*-Fe_2_O_3_.[19] In addition, previous investigations [22] on γ-Fe_2_O_3_/SiO_2_ nanocomposites heated at different temperatures have even evidenced the possibility to stabilize an ε-Fe_2_O_3_ phase. The ε-Fe_2_O_3_ phase has recently received much intention due to its very large coercive field value.[23,24] Previous studies have shown that this phase can be produced by plants [25] and formed from almandine garnets [26,27] and iron rich clays [28] when exposed to different high-temperature treatments. However, it is still difficult to synthesize ε-Fe_2_O_3_ as a single-phase sample since ε-Fe_2_O_3_ is considered to be an intermediate polymorph between maghemite and hematite.[10,29–31]

In this article, we report the effect of both laser irradiation and thermal treatment on the structural stability and phase transitions of γ-Fe_2_O_3_ NPs homogeneously dispersed in silica matrix. γ-Fe_2_O_3_ NPs of 4 ± 1 nm diameter have been prepared by co-precipitation and sol-gel methods and then randomly dispersed into an amorphous silica matrix with different mass fraction values (0.07 to 1). Raman spectroscopy is a very suitable technique for studying *in situ* laser irradiation effects. In particular, the laser used in the Raman system can play different roles: (i) a high intensity radiation source to induce phase transformation and (ii) an excitation source to characterize *in situ* the vibrational modes and thus the structural properties of the materials. Much attention was paid to the growth and presence of ε-Fe_2_O_3_ polymorph versus oxide concentration and in addition a structural diagram will be established for the γ-Fe_2_O_3_/SiO_2_ system versus laser irradiation power.

## 2. Experimental section

### 2.1. Synthesis of γ -Fe_2_O_3_ nanocrystals

γ-Fe_2_O_3_ NPs were prepared according to Massart’s method [32] with the cationic precursors used in the form of metallic salts soluble in water. The experimental procedure was detailed in our previous studies.[33] The co-precipitation method is a simple route of synthesizing maghemite and other ferrite NPs from ferric and ferrous salts. In addition, iron oxide nanocrystals with composition close to magnetite were first prepared [34,35] by soft chemistry using coprecipitation of the precursors cation FeSO_4_ .4H_2_O and Fe(NO_3_)_3_ .6H_2_O (Fe^2+^/Fe^3+^ = 1/2). Those nanocrystals were then oxidized with 2 M HNO_3_ and 0.33 M Fe(NO_3_)_3_ until a complete transformation into maghemite. After centrifugation, the nanocrystals were peptized in 2 M HNO_3_ to create positive surface charges. After stirring in a solution of 2 M nitric acid, the precipitate was washed several times with acetone and was finally peptized in pure water, to form a stable sol (pH ≈ 2) with minimum aggregation of nanocrystals.[36,37]

### 2.2. Synthesis of γ-Fe_2_O_3_ / SiO_2_ nanocomposites

The nanocomposites were prepared by using the sol-gel procedure described previously.[33,38,39] Maghemite solutions (called ferrofluid) must be prepared in advance before being dispersed in an amorphous silica matrix. The first step is to form a clear solution of molar ratio 1 TEOS (tetraethoxysilane):73C_2_H_5_OH:8.3H_2_O:0.01HCl. This solution was stirred during 15 min before adding the ferrofluid to the solution. The resulting solution was mixed in a steam bath for 48 h at 80 °C until obtaining a solid brown-colored sample.

Polymerized tetraethoxysilane (TEOS) network is often used as a surface coating material for iron oxide nanocrystals. Indeed, this coating can prevent aggregation in solution and improve the chemical stability. Additionally, polymerized silica-coated iron oxide nanocrystals exhibit good biocompatibility and solubility in water.

We differentiated the samples by mass fraction *ρ*_r_ that represents the weight of NPs in each sample:

$$\rho_{\text{r}}=mass(\gamma -Fe_{\text{2}}{\text{O}}_{\text{3}}\text{) / total mass}\text{.}$$

A sample, so-called *ρ*_r_ = 1, corresponds to bare maghemite NPs. The nano-composites were further heated in air up to 750, 850, 1000, 1200, and 1400 °C successively for 30 min and then cooled down to room temperature. Heating and cooling rates were set at 5 °C/min.

### 2.3. Structural analysis

X-ray powder diffraction (XRD) measurements were performed with a Siemens D500 setup, employing Cu-K*α*_1,2_ radiation (λ_1_ = 1.5406Å, λ_2_ = 1.5444 Å). The measurements were carried out in the 2θ range 20–100° with a step size of 0.02°. The crystallite parameters were determined using the MAUD [40] program. Rietveld fitting of the full pattern was combined with Fourier analysis to describe the broadening of peaks.[41]

The microstructures, size and distribution of nanocrystals in the silica matrix were inspected by a transmission electron microscope JEOL 2100 (JEOL, Tokyo, Japan) operated at 200 kV. Samples were prepared according to the following protocol: a small amount of sample was crushed in an agate mortar containing absolute ethanol. A drop of the suspension is then deposited on a copper grid covered with amorphous carbon membrane holes. The grid was dried and then inserted into the TEM for measurements.

### 2.4. Laser irradiation and Raman analysis

The Raman spectra were recorded at room temperature in the backscattering configuration of a T64000 (Jobin-Yvon, Horiba Group, Kyoto, Japan) spectrometer under a microscope with a 100× objective focusing the 514 nm line from an argon–krypton ion laser (coherent, Innova). The spot size of the laser was estimated to 0.8 μm. Measurements using different laser output powers between 1 and 600 mW (corresponding to a laser power of approximatively 0.08–50 mW at the sample) were carried out. The Raman spectra were systematically recorded twice with an integration time varying between 400 and 600 s. Acquisition and basic treatments of spectra have been made with the LabSpec V5.25 (Jobin-Yvon, Horiba Group, Kyoto, Japan) software. Data analysis and Gaussian curve-fitting procedures of spectra have been made using OriginPro 8.6 software (OriginLab Corporation, Northampton, MA, USA).

## 3. Results

### 3.1. Structural characterization

**Figure 1.**
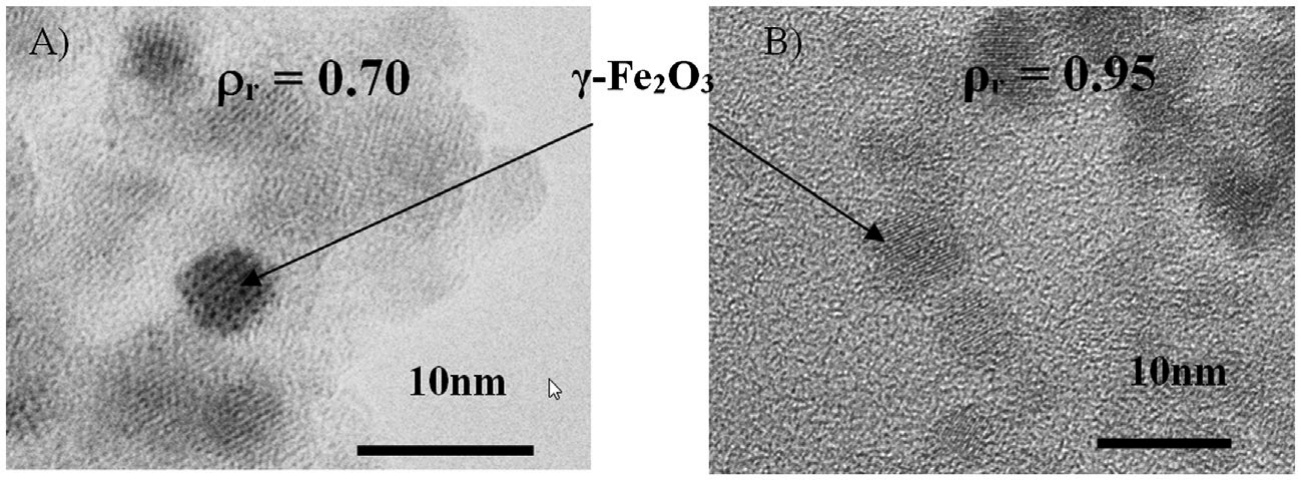
(a) TEM micrograph of γ-Fe_2_O_3_/SiO_2_ composites *ρ*_r_ = 0.70, (b) *ρ*_r_ = 0.95.

Figure 1 shows typical TEM images of two as-prepared nanocomposites *ρ*_r_ = 0.70 and 0.95. The contrast between Fe_2_O_3_ and SiO_2_ is evidenced, the silica matrix as the lighter background and the maghemite NPs with darker contrast. The silica matrix causes steric repulsion between NPs and prevents them from aggregating. Since the NPs are well dispersed even for high *ρ*_r_ values, images of individual NPs can be obtained. The images reveal that the NPs are crystallized, relatively spherical and monodisperse with a size distribution between 3 and 5 nm. Their average diameter <D_MET_> estimated from several TEM images was then confirmed by XRD analysis.

**Figure 2.**
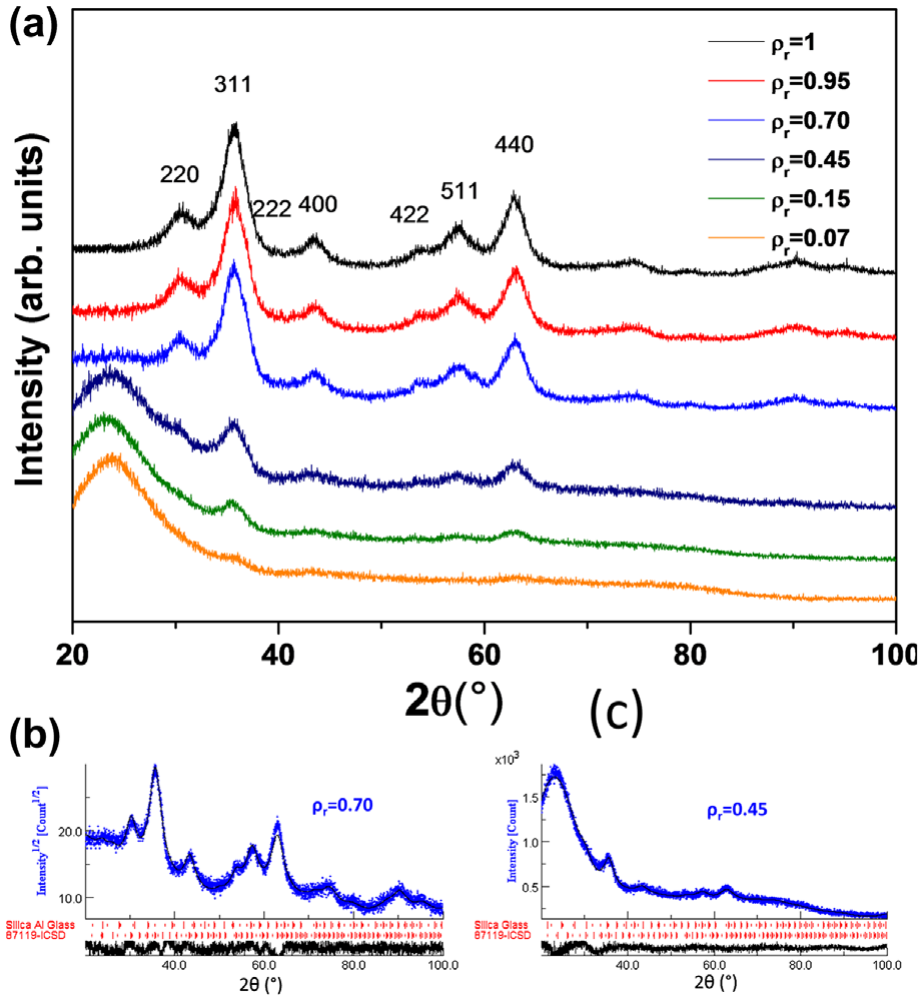
(a) XRD patterns of γ-Fe_2_O_3_/SiO_2_ nanocomposites with *ρ*_r_ = 0.07; 0.15; 0.45; 0.70; 0.95 and 1. Refinement of the diffraction patterns of two nanocomposites; (b) *ρ*_r_ = 0.70 and (c) *ρ*_r_ = 0.45. The diffraction peaks of maghemite are identified by the corresponding Miller indices.

The powder X-ray diffraction patterns of as-prepared mesoporous silica samples with different concentrations of NPs are presented in Figure 2(a), while the refined diffraction patterns for *ρ*_r_ = 0.70 and *ρ*_r_ = 0.45 are illustrated in Figure 2(b) and 2(c), respectively. The diagrams for nanocomposites with low concentrations ranging from 0.07 to 0.45 consist of a prominent broad peak around 22° corresponding to amorphous silica matrix. The major diffraction peak at 35.5° (311) in addition to minor peaks at 30.5° (220), 43.5° (400), 57.5° (511), and 63° (440) confirm the spinel structure of iron oxide maghemite.[13,14,33] When the concentration of NPs increases above *ρ*_r_ = 0.70, the broad peak associated with silica disappears and the diffraction peaks assigned to 220, 311, 222, 400, 422, 511, and 440 reflections of the cubic spinel structure of maghemite (JCPDS 16–629) are better observed. Moreover, a previous study by ^57^Fe Mössbauer spectrometry has shown that the hyperfine structure can be well described by means of a distribution of quadrupolar doublets and the mean values of the isomer shift are characteristic of ferric species Fe^3+^ which fully confirmed the presence of maghemite.[33]

Table 1 summarizes the refinement parameters for as-prepared γ-Fe_2_O_3_@SiO_2_ composites with different *ρ*_r_ values. The calculated lattice parameters of maghemite nanocrystals are very similar for all nanocomposites. The agreement factor values (R_exp_, R_p_ and χ^2^) obtained by MAUD show the good quality of the refinement. As expected, the average diameters of maghemite NPs, which agree well with TEM size distributions, do not depend on the *ρ*_r_ values. The lattice parameter for the cubic spinel structure was found intermediate between those of bulk maghemite (0.834 nm) and bulk magnetite (0.839 nm). The values of microstrain remain low for all the nanocomposites.

**Table 1.** Refined values of lattice parameter of as-prepared γ-Fe_2_O_3_/SiO_2_ composites for different mass fractions *ρ*_r_ = 0.07, 0.15, 0.45, 0.7, 0.95 and 1. The used fitting method allows one to determine the coherence domain length mean value defined as apparent crystallite size 〈D〉 and the root mean square (Rms) microstrain value and the corresponding agreement factors of as-prepared maghemite powders.

	Phase	a (nm)	<D> (nm)	Rms microstrains (10^−4^)	R_exp_	R_w_	χ_i_^2^
*ρ*_r_ = 1	γ	0.836 ( ± 0.002)	4 ( ± 1)	2.9 ( ± 0.5)	11.74	8.05	1.77
*ρ*_r_ = 0.95	γ	0.836 ( ± 0.002)	4 ( ± 1)	8.0 ( ± 0.5)	11.45	9.21	1.54
*ρ*_r_ = 0.70	γ	0.835 ( ± 0.002)	4 ( ± 1)	23 ( ± 5)	8.73	6.82	1.64
*ρ*_r_ = 0.45	γ	0.835 ( ± 0.002)	4 ( ± 1)	57 ( ± 5)	6.75	5.89	1.31
*ρ*_r_ = 0.15	γ	0.835 ( ± 0.002)	4 ( ± 1)	10 ( ± 2)	5.21	4.39	1.40
*ρ*_r_ = 0.07	γ	0.834 ( ± 0.002)	4 ( ± 1)	10 ( ± 2)	5.24	4.88	1.53

**Figure 3.**
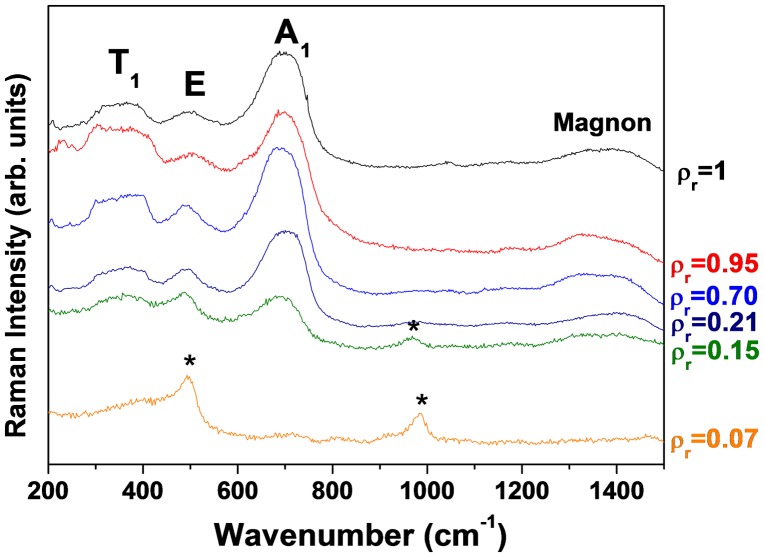
Raman spectra of γ-Fe_2_O_3_ composites with *ρ*_r_ = 0.07, 0.15, 0.21, 0.70, 0.95, 1 recorded at an output laser power of 5 mW, λ = 514.5 nm. *The characteristic vibration bands of the silica.

Raman spectroscopy is a convenient tool to distinguish iron oxides and in particular to differentiate maghemite and magnetite, since the modes of vibration characteristics of maghemite can easily be localized in spite of their large width.[42,43]. Therefore, for the cubic spinel structures, five Raman active modes are expected (A_1_ + E + 3T_1_). In the case of the maghemite, the Raman spectra are directly related to the degree of crystallinity,[44] and thus the lack of ion exposes it only to three large bands localized at ≈ 700 cm^−1^ (A_1_), ≈ 500 cm^−1^ (E) and ≈ 350 cm^−1^ (T_1_). The Raman spectra of as-prepared γ-Fe_2_O_3_/SiO_2_ with different mass fractions of γ-Fe_2_O_3_ (*ρ*_r_ between 0.07 and 1) are shown in Figure 3. The spectra recorded at a laser power of 5 mW exhibit the three broad bands characteristic of maghemite in addition to a broad band near 1400 cm^−1^ attributed to the magnon mode of γ-Fe_2_O_3_.[13] The Raman vibrational bands (indicated with asterisks) near 500 and 1000 cm^−1^ are characteristic of the Si-O-Si stretching modes of the silica matrix.[19] The intensity of these modes gradually decreases with the increase of iron oxide mass fraction (*ρ*_r_ = 0.70). Such behavior is similar to that observed by X-ray diffraction with the broad peak corresponding to amorphous silica matrix.

### 3.2. Effect of laser power

Recently, we have shown that during micro-Raman measurements (with a laser light focused to a small volume) it is possible to unintentionally heat a sample with a few milliwatts laser power.[13,14] We first evidenced this phenomenon on maghemite NPs at low laser power (from 1 mW) when irreversible structural changes occur. Then we confirmed that when the laser power is increased the transition from γ to *α* can take place immediately due to a localized heating effect. This transition is accompanied by a progressive increase of the nanoparticle sizes with a reduction of the microstrain. This result was also confirmed by Raman analysis based on the positions and widths of bands. More recently, it has been reported that the surface coating of maghemite NPs with organic molecules helped both in reducing the number of surface defects of NPs and in stabilizing thermally the γ-Fe_2_O_3_ NPs. Consequently, the maghemite–hematite phase transition is temperature delayed.[12,15]

**Figure 4.**
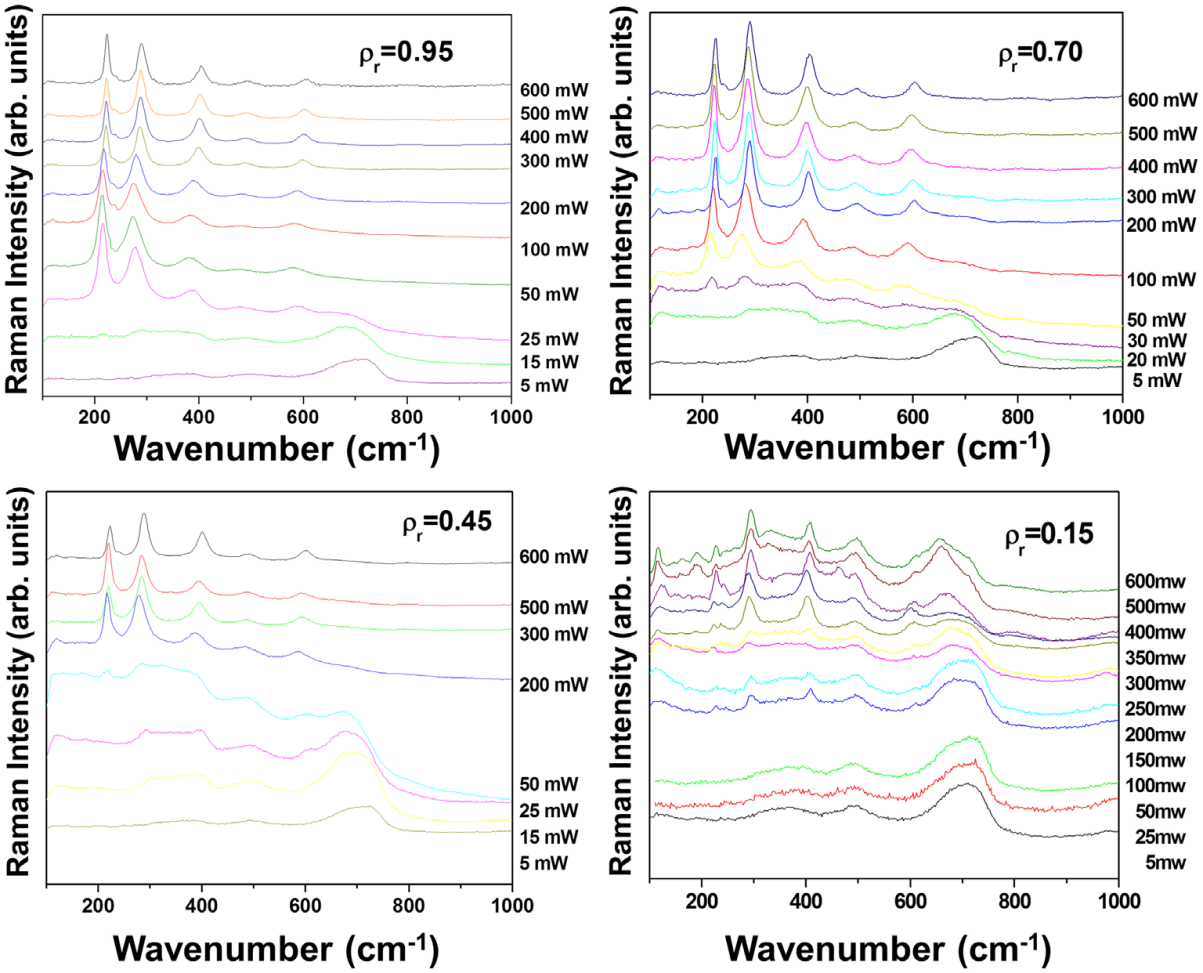
Raman spectra of samples with *ρ*_r_ = 0.95, 0.70, 0.45 and 0.15, recorded at laser powers between 5 and 600 mW.

Raman spectra of γ-Fe_2_O_3_/SiO_2_ nanocomposites with different mass fractions *ρ*_r_ (0.95, 0.70, 0.45 and 0.15) versus the laser power ranging from 5 to 600 mW are illustrated in Figure 4. It can be observed that the laser power necessary to induce the transformation of maghemite to hematite depends on the mass fraction *ρ*_r_. The thermal behavior of the sample *ρ*_r_ = 0.95 is very similar to that of pure maghemite studied previously.[14] However, for the low concentration, the behavior differs greatly. In fact, for concentration less than 0.45, the analysis of Raman spectra shows that up to 600 mW, no hematite vibration mode is observed. This different behavior at low (*ρ*_r_ < 0.45) and high concentrations (*ρ*_r_ ≥ 0.45) reveals that silica prevents the transformation from maghemite to *α*-Fe_2_O_3_ NPs.

If we focus now on the larger A_1_ (≈225 cm^−1^) and 2E_g_ (≈290 cm^−1^) vibrational modes of hematite, one can notice that the frequency position, linewidth and intensity of these bands strongly depend on *ρ*_r_. For high concentration values, the maghemite starts to transform into hematite between 15 and 30 mW while the widths of the vibrational modes decrease when the laser power increases. Such a behavior can be explained by a progressive crystallization of hematite to a polycrystalline phase. In the case of low concentrated nanocomposites, maghemite can be observed up to a power of 300 mW and when the laser power increases, the Raman spectra are difficult to analyze because new broad bands in the range 100–800 cm^−1^ appear, juxtaposed with a luminescence signal. At this stage, these vibration modes have not been clearly identified; however, since they are observed in the vicinity of 200–800 cm^−1^ range they can probably be attributed to metastable iron oxide phases.[19,45] Sartoratto et al. [19] reported the set of three Raman bands, peaking at 240, 310 and 428 cm^−1^, which may be attributed to the Fe–O stretching modes of the ε-Fe_2_O_3_ phase. Moreover, as ε-Fe_2_O_3_ is considered as an intermediate phase between the maghemite and hematite,[19,45–47], it is quite likely that among the structural transformations induced under laser irradiation the vibrational signature of the metastable ε-Fe_2_O_3_ phase appears.

As previously reported,[13,14] the mechanism of the thermally induced polymorphous transformation (after heat treatments in a furnace and under laser irradiation) can be studied from the analysis of the Raman spectra using: (i) a baseline profile analysis approach based on the difference between the intensity of two reference-like lines L_MIN_ and L_MAX_ centered approximatively at 340 and 850 cm^−1^; and (ii) the evolution of the wavenumber and bandwidths of the stronger A_1_ (~225 cm^−1^) and 2E_g_ (~290 cm^−1^) vibrational modes of hematite phase under laser irradiation.

#### 3.2.1. (I(L_MIN_) – I(L_MAX_)) evolution

**Figure 5.**
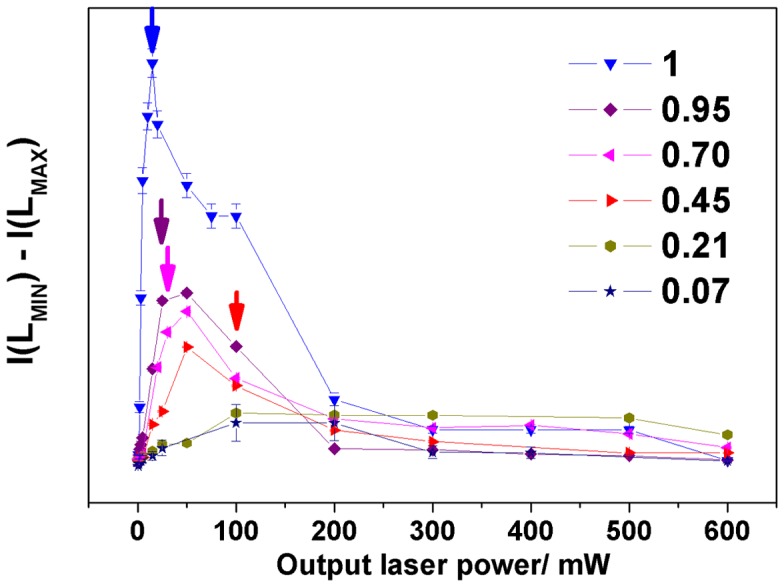
Evolution of the calculated (I(L_MIN_) – I(L_MAX_)) intensity versus nominal output laser power. The position of the arrows indicates the output laser power where it has been possible to detect the vibrational bands characteristic of hematite.

A previous Raman investigation on maghemite NPs (*ρ*_r_ = 1) revealed that the evolution of (I(L_MIN_) – I(L_MAX_)) is directly correlated to structural and morphological modification of NPs.[14] Figure 5 illustrates the evolution of calculated intensity (I (L_MIN_) – I (L_MAX_)) for the γ-Fe_2_O_3_/SiO_2_ nanocomposites with different mass fractions *ρ*_r_ versus the nominal output laser power. At low laser power and below 20 mW, the significant increase of this intensity value suggests that under a microscope the laser energy density induces immediately a rise of temperature that is sufficient to cause structural modifications at the surface of maghemite NPs. Note that the observation of vibrational bands characteristic of hematite has been systematically reported (Figure 5) a few milliwatts below the maximum of the curves (at 15 mW for *ρ*_r_ = 1, 20 mW for *ρ*_r_ = 0.95, 30 mW for *ρ*_r_ = 0.70 and 100 mW for *ρ*_r_ = 0.45). This maximum shifts to high values of laser power for mass fraction *ρ*_r_ ranging from 0.95 to 0.45.

These differences can be mainly explained by the stabilization of the maghemite NPs when they are embedded into silica matrix: the more the NPs are dispersed the more laser energy density is needed to aggregate NPs together to initiate the coalescence process (according to the interpretation observed previously on uncoated and as-prepared powdered maghemite NPs).[14] The presence of the silica matrix acts for the maghemite NPs as a coating that limits both the agglomeration of NPs, prevents the process of particle growth and therefore delays the γ-Fe_2_O_3_ to *α*-Fe_2_O_3_ phase transition. The decrease in values (I (L_MIN_) – I (L_MAX_)) between 500 and 600 mW for nanocomposites with high mass fraction can be explained by a progressive crystallization of particles and the increase of grain sizes in a polycrystalline state.[13] On the other hand, it was also shown that when the evolution of the intensity of the baseline (I (LMIN) – I (LMAX)) is small, it suggests that NPs undergo only slight structural changes at the surface under irradiation.[14] Accordingly, for mass fraction below *ρ*_r_ = 0.21, and for small laser power, our results reveal that laser irradiation stabilize somehow the NPs compared to NPs incorporated into nanocomposites with high mass fraction. However, no information about the stabilized phases can be deduced from the curve features.

#### 3.2.2. Wavenumber analysis

The shift of Raman vibrational modes of hematite as a function of laser power has been attributed mainly to two effects:[12,13,44,48] thermal expansion (or volume change) and stresses in the structure resulting from the increasing local temperature. In recent studies [13,14] we have shown that phonon confinement, strain, size distribution, defects and variations in phonon relaxation with particle size contribute to the changes in the Raman peak position and linewidth as the laser power increases.

**Figure 6.**
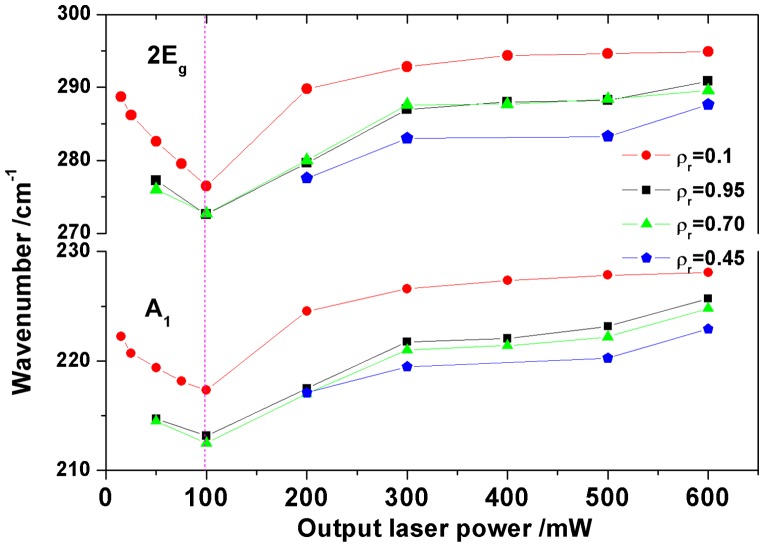
Laser power dependence of the wavenumber of A_1_ (~225 cm^−1^) and 2E_g_ (~290 cm^−1^) modes of hematite (*ρ*_r_ = 0.45, 0.70, 0.95 and 1).

Figure 6 illustrates the evolution of the frequency of the two strong vibration modes A_1_ and 2E_g_ of hematite phase versus the nominal output laser power. The appearance of these modes was clearly observed after laser irradiation on as-prepared powdered γ-Fe_2_O_3_ NPs and on three different γ-Fe_2_O_3_/SiO_2_ nanocomposites with mass fractions *ρ*_r_ = 0.95, 0.70 and 0.45. This analysis was also undertaken for lower mass fractions; however, it was not possible to assign distinctly hematite vibrational bands.

So, in Figure 6, between 15 and 100 mW, the Raman spectra suggest the coexistence of maghemite and hematite and the analysis of the lineshape of A_1_ and 2E_g_ modes reveal a shift towards lower wavenumber. For as-prepared maghemite powder, this shift was previously attributed to stress effects and the creation of defects on the NPs surface resulting from a sudden increase in local temperature. For *ρ*_r_ = 0.95 and *ρ*_r_ = 0.70, the frequency variation is calculated as 2 and 5 cm^−1^ for 50 to 100 mW, respectively. At 100 to 600 mW, progressive shifts to higher frequencies were then observed, indicating structural relaxation phenomena associated with a long-range reorganization. As the frequency behaviors were similar to those observed for maghemite powder under irradiation, these results suggest the existence of the same effects but with a probable less pronounced increment size in nanocomposites. This assumption is in good agreement with the conclusions obtained by Jubb and Allen:[44] indeed, they observed that the 250 nm thick hematite film exhibits both blue-shifted frequencies (namely the Raman peaks shift to higher wavenumber side) and broader FWHM of phonon peaks compared to the 50 nm thick film.

For mass fraction *ρ*_r_ = 0.45, the weak bands attributed to hematite might be first observed at 100 mW and unambiguously at 200 mW (Figure 4). As can be observed in Figure 6, this nominal laser power value corresponds to the lower frequencies for higher mass fraction (due to the germination of the hematite phase at the nanoparticle interfaces); in addition, this laser power corresponds to that necessary to initialize a coalescence process and crystallization of hematite particles, as previously reported.[14] Therefore, at this particular mass fraction, the coexistence of maghemite and hematite is minimal and the Raman spectra reveal above 100 mW similar frequency behaviors (to those already observed for more concentrated composites) since it results from an increase of particle size.

When we compare the frequency values of the vibration modes A_1_ and 2E_g_ at 600 mW for nanocomposites with those of irradiated as-prepared maghemite NPs (228 and 295 cm^−1^), we can observe that for *ρ*_r_ = 0.95 and *ρ*_r_ = 0.70 nanocomposites the values (226 and 290 cm^−1^) correspond to those recorded at 200 mW on maghemite powder and even below if we compare to the higher value measured for *ρ*_r_ = 0.45 (223 and 288 cm^−1^). As we previously established, the correlation between the effects induced by the output laser power and the temperature of thermal treatment for γ-Fe_2_O_3_ and *α*-Fe_2_O_3_ phases,[14] the local temperature of the sample heated under laser excitation and the average grain size of hematite particles can be estimated. We previously established that an output laser power of 150 mW and 200 mW correspond to heat the powder to approximatively 400 and 500 °C and to form particles with an average diameter of about 18 and 35 nm, respectively. But, at 600 mW it corresponds to 800 °C with particles of around 130 nm diameter.

#### 3.2.3. Evolution of bandwidths

**Figure 7.**
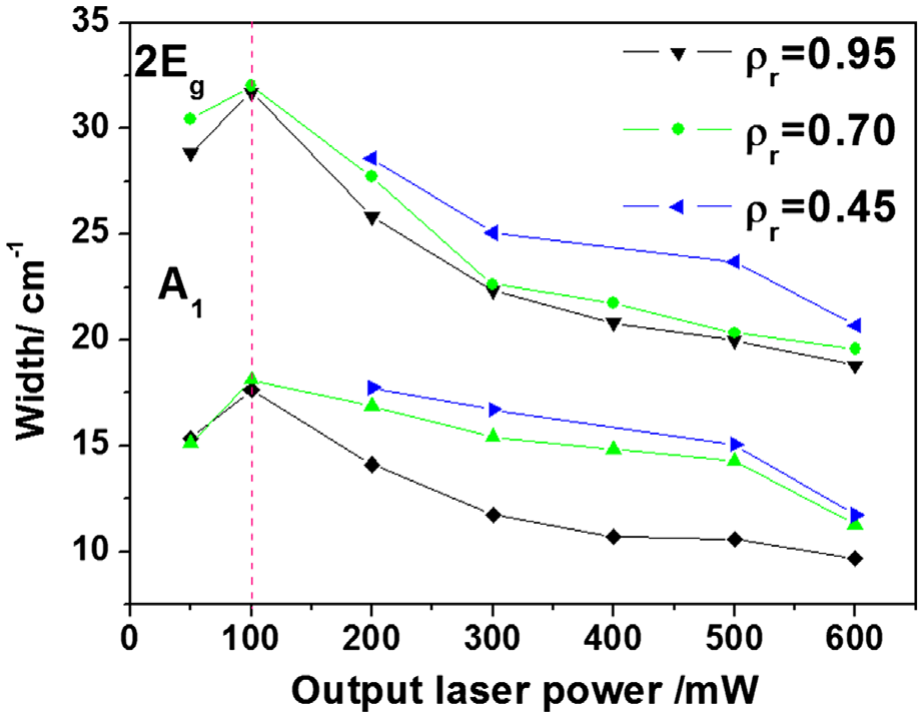
Laser power dependence of the widths of A_1_ (~225 cm^−1^) and 2E_g_ (~290 cm^−1^) hematite modes for three nanocomposites (*ρ*_r_ = 0.95, 0.70 and 0.45).

Figure 7 describes the evolution of the widths of hematite bands (A_1_ and 2E_g_ modes) for three nanocomposites (*ρ*_r_ = 0.95, 0.70 and 0.45) as a function of the nominal output laser power. The behavior of the bandwidth presents two regimes: one before 100 mW, and the second one above 100 mW. From the appearance of A_1_ and 2E_g_ modes on the Raman spectra (i.e. with the coexistence of maghemite and hematite) up to 100 mW an increase of bandwidths is observed. This behavior is likely due to stress effects on the surface of the nanocrystals as previously mentioned.[13,14] Above 100 mW, the shift of 2E_g_ and A_1_ modes towards higher frequencies is accompanied with a significant decrease of the bandwidths. This feature can be mainly explained by both a structural relaxation phenomenon and a progressive crystallization of hematite particles accompanied by an increase in grain size.[14] It is also important to emphasize from Figure 7 that at a given laser power, the bandwidth of the A_1_ and 2E_g_ modes decreases with the iron oxide concentration.

## 4. Discussion

In our recent studies,[13,14] we have demonstrated that the structural modifications induced on γ-Fe_2_O_3_ NPs under laser irradiation and the γ-Fe_2_O_3_ to *α*-Fe_2_O_3_ phase transition result from a thermal effect associated with an increase in the grain sizes. Recently, our investigations on coated γ-Fe_2_O_3_ NPs with oleic acid and oleylamine molecules [15] confirm that the laser power density (and thus temperature) required to induce the γ to *α*-Fe_2_O_3_ phase transition depends on both the size and the surface state of NPs. In the present study, we investigate the thermal effect of the laser irradiation on NPs homogeneously dispersed in silica matrix. As already mentioned, our results confirm that the silica matrix acts as a coating that limits both the agglomeration of NPs, prevents the process of particle growth and therefore delays the γ-Fe_2_O_3_ to *α*-Fe_2_O_3_ phase transition. In this particular environment, as it was previously shown that it is possible to enhance the stability of NPs,[49] we thus decided to initiate metastable phases under laser irradiation and then study by micro-Raman spectroscopy the formation of ε-Fe_2_O_3_ phase. Indeed, by taking advantages of the dispersion of NPs (limiting the coalescence process between adjacent NPs), structural modifications can be induced easily at the surface of the γ-Fe_2_O_3_ NPs under laser irradiation even at low energy density. Moreover, with the presence of oxygen atoms surrounding the NPs, we should be able to both initiate and stabilize the metastable ε-Fe_2_O_3_ phase and thus to determine its characteristic Raman spectrum.

### 4.1. Formation of ε-Fe_2_O_3_ phase by heat treatment of γ-Fe_2_O_3_/SiO_2_ nanocomposites

**Figure 8.**
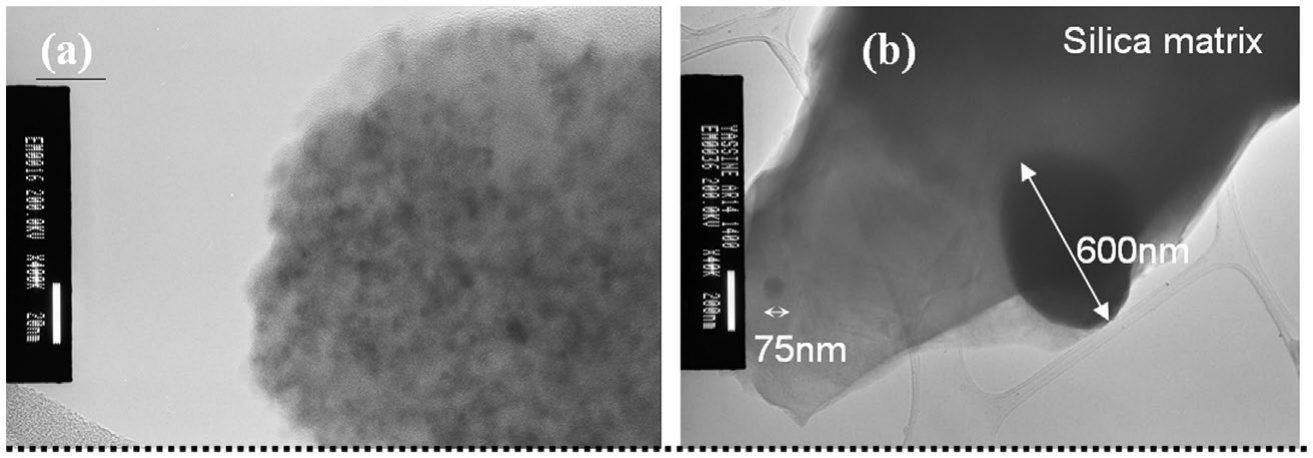
TEM micrographs of the nanocomposite *ρ*_r_ = 0.15; (a) at room temperature (scale bar 20 nm), (b) at 1400°C (scale bar 200 nm).

Preliminarily and in order to observe the annealing effect on structural properties of γ-Fe_2_O_3_ NPs dispersed into silica matrix, we performed TEM analysis on nanocomposite with low concentration *ρ*_r_ = 0.15 before and after annealing at 1400 °C (Figure 8). Our treatment consists in annealing isothermally at 1400 °C for 30 min with a heating rate and cooling of 5 °C min^–1^. It can be noticed in Figure 8 that the thermal annealing originates growth of nanocrystals with nanometric dimensions (75 nm in Figure 8) for NPs still trapped into the silica matrix and larger particles with dimensions greater than 200 nm when the silica matrix is sintered.[50] The increase in NPs size is likely due to the nanocrystals coalescence by diffusion of iron cations.[44] Indeed, it was concluded that the annealing treatment promotes the atomic diffusion and generates an increase in size, a reduction of point defects and, therefore, a better crystallinity of the particles. These results are in good agreement with those obtained by Alcalá and Real [51] and Taboada and Gich.[52] Indeed, they also concluded that the magnification of the particle size depends on the structural properties of silica matrix. So, in the vicinity of the glass transition temperature of the silica in the range of 800–1200 °C, maghemite nanocrystals may come into contact, coalesce and easily convert into other phases of iron oxides (if the density of NPs is high enough). In other words, the sintering of the mesoporous silica matrix can be seen in our case as a mechanical consolidation process which leads to an extensive agglomeration of NPs (initially dispersed) and grain growth.

**Figure 9.**
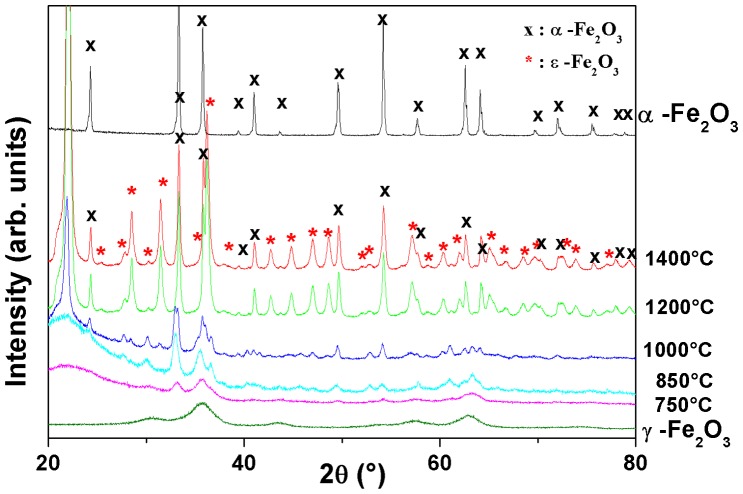
XRD patterns of γ-Fe_2_O_3_@SiO_2_ nanocomposites (*ρ*_r_ = 0.15) annealed at different temperatures. The diffraction lines of ε-Fe_2_O_3_ phase are identified. The XRD patterns of maghemite and hematite are presented for comparison.

The X-ray diffractograms of the nanocomposites annealed at 25–700 °C (Figure 9) indicate that maghemite remains stable up to 700 °C.[53] At 750–850 °C, two phases can be unambiguously identified, i.e. γ-Fe_2_O_3_ and ε-Fe_2_O_3_: indeed, the presence of diffraction peaks (220, 311, 222, 400, 422, 511, 440) are characteristic of cubic face-centered structured γ-Fe_2_O_3_; and the diffraction peaks (022, 013, 113, 200, 201, 130, 131, 132, 212, 133, 204, 134, 144, 135, 330, 126, 252) correspond to those of the ε–phase with an orthorhombic crystal structure (SG: P*na*2_1_) and with lattice parameters a = 5.08 Ǻ, b = 8.78 Ǻ, c = 9.47 Ǻ, and *α* = *β* = *γ* = 90°.[51,54] We also notice that the mean crystallite size of the iron oxide NPs, as estimated from the broadening of the diffraction peaks, increases with the annealing temperature mainly up to 1000 °C and above; its main effect is then to increase the crystallinity of the NPs. At 1000 °C, the diffraction peaks of maghemite vanished and all peaks in the range 23–100° can then be assigned to both *α*-Fe_2_O_3_ and ε-Fe_2_O_3_. This result has been then confirmed by ^57^Fe Mössbauer spectrometry (Figure S1). The broad peak around 22° corresponding to amorphous silica matrix has been transformed into a strong diffraction peak around 21.8°, demonstrating the crystallization of the amorphous silica into cristobalite.[52]

Between 1000 and 1400 °C, we can observe a decrease of the ε-Fe_2_O_3_ phase and an increase of *α*-Fe_2_O_3_ peaks, resulting in an increase in the amount of hematite as the temperature increases. Accordingly, TEM and XRD results show that the transformation of γ-Fe_2_O_3_ and ε-Fe_2_O_3_ to the stable iron oxide *α*-Fe_2_O_3_ is triggered by the devitrification–crystallization of the silica matrix. Such a result points out the prominent role of the physicochemical properties of the silica matrix in the formation and stability of successive iron oxide phases. The thermal stability of γ-Fe_2_O_3_ is increased up to about 750 °C by the presence of the silica amorphous matrix, while in the absence of the latter, the maghemite to hematite phase transformation is reported within the temperature range 400 °C depending on the crystallinity and the particle size, i.e. the preparation method, preparation conditions, etc.[10–14]

The metastable ε-Fe_2_O_3_ phase is known to be difficult to grow and to obtain as a single phase. In addition, it consists of an intermediate phase of maghemite γ-Fe_2_O_3_ and hematite *α*-Fe_2_O_3_, ε-Fe_2_O_3_ is generally detected together with γ and/or *α* polymorphs. Moreover, to our knowledge ε-Fe_2_O_3_ has been currently synthesized in nanosized forms [55–57] and no micrometer-sized crystals have been reported yet. This feature has been interpreted by a low surface energy which also suggests that the formation of ε-Fe_2_O_3_ requires a certain rate of agglomeration of γ-Fe_2_O_3_ precursor NPs,[22] generally achieved through the use of an insulating medium. In our case, the amorphous silica matrix formed from tetraethoxysilane (TEOS), provides a flexible and mesoporous medium that serves both as nucleation sites for the formation of ε-Fe_2_O_3_ nano-objects and as size-restricted spaces that prevent excessive particle agglomeration (as in powders) to form *α*-Fe_2_O_3_.[10,22]

**Figure 10.**
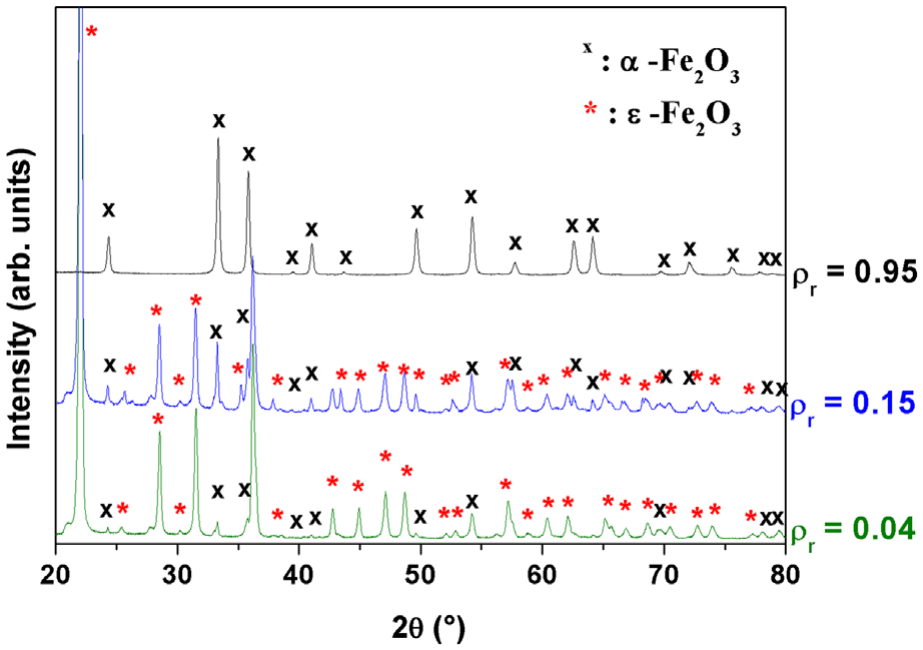
XRD patterns of the nanocomposites *ρ*_r_ = 0.95, *ρ*_r_ = 0.15 and *ρ*_r_ = 0.04 treated at 1400°C.

In order to better understand the effect of concentration on the phase transition for such composites, we performed XRD analysis of three nanocomposites annealed at 1400 °C: a nanocomposite with high concentration (*ρ*_r_ = 0.95) of γ-Fe_2_O_3_ NPs and two composites with low concentration (*ρ*_r_ = 0.04 and *ρ*_r_ = 0.15). In Figure 10, the diffraction pattern of the high concentrated nanocomposite shows reflections corresponding to polycrystalline hematite phase, as it was observed by heating pure maghemite powder. The hematite particles average diameter is evaluated to be 123 ± 10 nm. In the case of nanocomposites with low concentrations, the diffraction pattern confirms the existence of *α*-Fe_2_O_3_ phase and ε-Fe_2_O_3_ nanoparticles (Table S2). Microanalysis of X-rays diagram (Figure S2, Table S2) shows that the proportion of hematite increases with iron oxide concentration (47% of *α*-Fe_2_O_3_ for *ρ*_r_ = 0.04 and 64% for *ρ*_r_ = 0.15).

**Figure 11.**
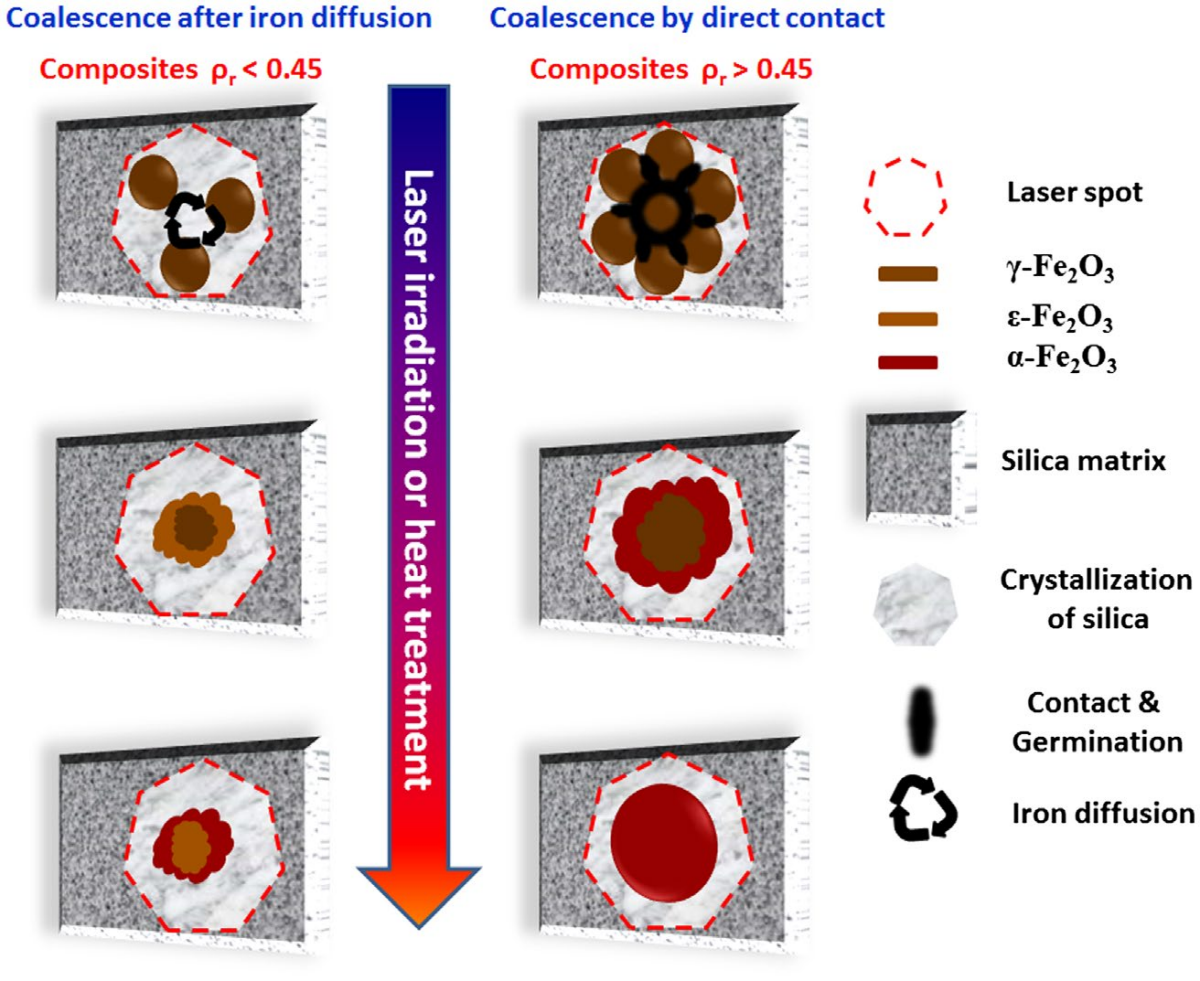
Sketch of the mechanism for the γ → ε → *α*-Fe_2_O_3_ phase transition occurring at the nanoscale under laser irradiation and heat treatment.

This study confirms that maghemite nanocrystals dispersed into a silica matrix transform into hematite by the above-mentioned heat treatment, and in the case of nanocomposite the mechanism related to this phase transition is also governed by the chemical properties of the host matrix. We also confirm that the phase transition into ε-Fe_2_O_3_ nanocomposites can be achieved by heat treatment in air for samples made of maghemite NPs dispersed into silica matrix with low iron oxide concentrations.[16] If NPs aggregation is high enough larger particles can be grown. The energy barrier impeding *α*-Fe_2_O_3_ formation is then overcome and ε-Fe_2_O_3_ is no longer favored. However, even if the annealing temperature is high enough to supply the activation energy needed for γ → ε → *α*-Fe_2_O_3_ transformation, the hematite phase cannot always be formed as the single phase for composites because the particle growth is impeded mainly by the dispersion of NPs over long distances. This explains why at 1400 °C, the transition into *α*-Fe_2_O_3_ is not complete for nanocomposites with *ρ*_r_ = 0.15 and *ρ*_r_ = 0.04 and how it has been possible to stabilize the ε-Fe_2_O_3_ phase over a wide temperature range; the amorphous silica matrix acts as a physical barrier limiting the diffusion of iron oxide (Figure 11).

### 4.2. ε-Fe_2_O_3_ phase induced under laser irradiation

Recently, Stagi et al. [58] studied the phase stability under laser irradiation of pure γ-Fe_2_O_3_ NPs and SiO_2_-embedded γ-Fe_2_O_3_ NPs. They confirmed that phase transformation of pure maghemite phase into hematite occurred at very low power densities. They concluded that the coalescence mechanism requires a close contact between iron oxide NPs, as reported by El Mendili et al. [13–15] for coated and uncoated NPs.^[13], [14], [15]^ They showed surprisingly that this phase transformation cannot be obtained by laser irradiation in the case of γ-Fe_2_O_3_–SiO_2_ core/shell system, but only by heat treatment at high temperature (1100 °C). In addition, these authors did not observe ε-Fe_2_O_3_ as an intermediate phase between maghemite γ-Fe_2_O_3_ and hematite *α*-Fe_2_O_3_. As already noticed in our studies, such a result is probably due to the high density of NPs (compared to the silica matrix), which easily favors an extensive agglomeration of NPs and then their growth.

**Figure 12.**
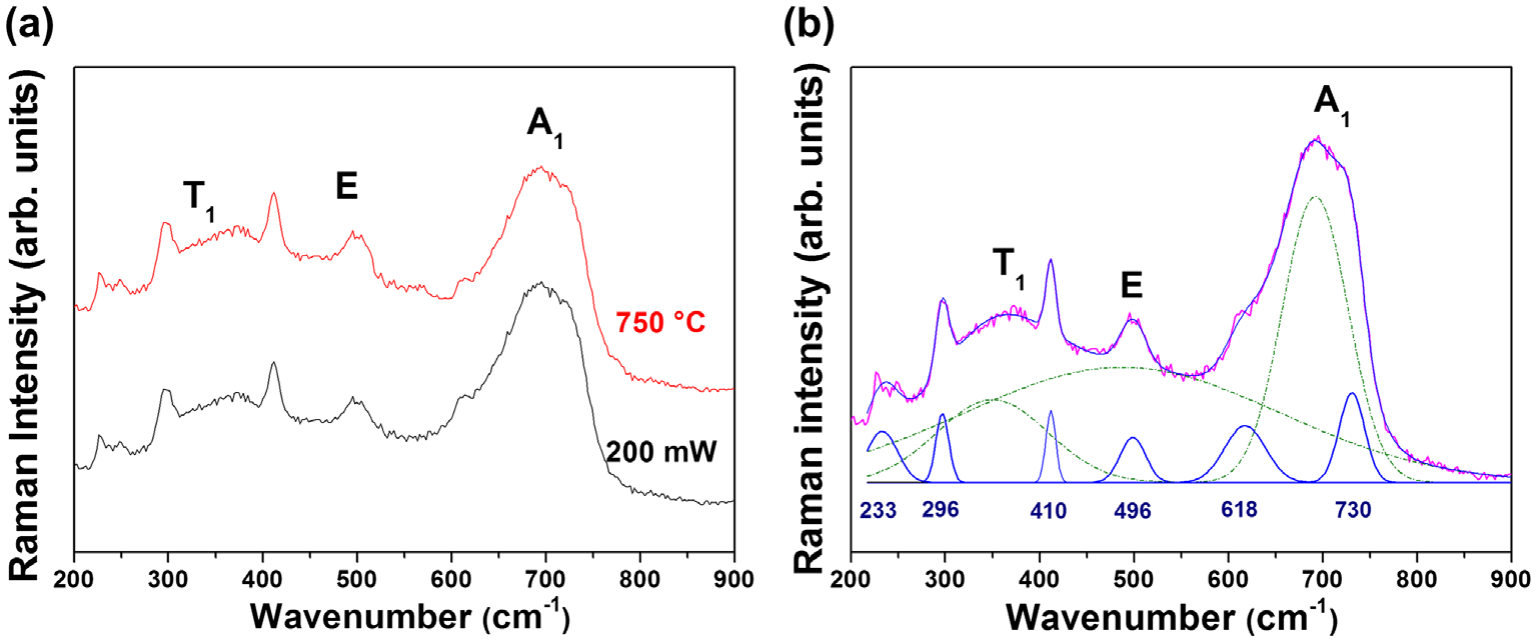
(a) Raman spectra of the sample *ρ*_r_ = 0.15, under irradiation with a laser power of 200 mW and that annealed at 750°C (recorded with an output laser at 5 mW); (b) Raman spectrum of the composite (*ρ*_r_ = 0.15) recorded at 200 mW with curve fitting results (the green bands present the maghemite vibration modes and the dark blue vibration bands present the new bands).

In our study, as previously illustrated in Figure 4, the Raman spectra recorded from 200 to 600 mW for the sample *ρ*_r_ = 0.15 were characteristic of a mixture of maghemite or hematite and another metastable iron oxide phases. Considering both the Raman spectrum of the sample annealed at 750 °C (Figure 12) and the XRD analysis (which confirmed the coexistence of both ε and γ phases), we can unambiguously confirm the formation of ε-Fe_2_O_3_ phase under laser irradiation for the nanocomposite recorded at 200 mW. Indeed, in Figure 12, the spectra show a similar profile in the wavenumber 200–800 cm^−1^ range, with the presence of three strong and broad bands at 350, 500 and 700 cm^−1^ commonly attributed to maghemite vibration modes and additional bands located around 233, 296, 410, 496, 618 and 730 cm^−1^ which may be attributed to ε-Fe_2_O_3_.

**Figure 13.**
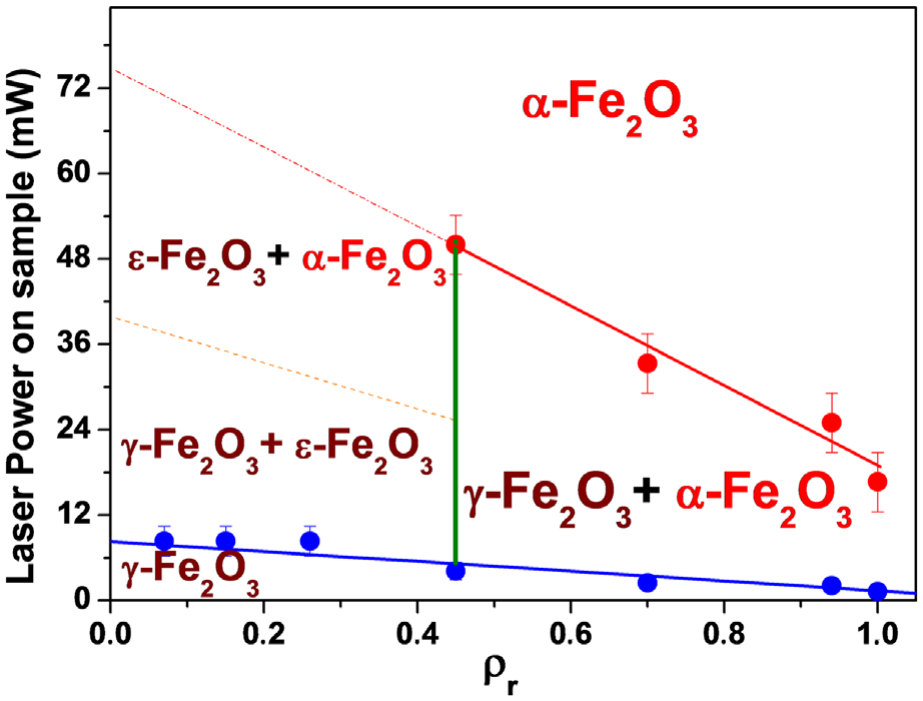
Structural behavior of γ-Fe_2_O_3_ nanocrystals dispersed in porous silica matrix under laser irradiation as function of excitation power on sample and of *ρ*_r_.

So, we summarized in Figure 13 the structural evolution of the composites as a function of both concentration *ρ*_r_ and energy density delivered by the laser irradiation at the sample (under the objective). When the laser output power is about 600 mW, the laser energy density delivered to the sample can be estimated (in a volume close to 1 μm^3^) to be approximately 50 GW cm^–3^. Phase separation was performed in Raman spectra by the detection of vibration modes in accordance with the data obtained by XRD analysis of nanocomposites heated at different temperatures.

In Figure 13, the blue line represents the first transition, where new Raman bands (different from that of maghemite) appear on the spectrum. The green line (at *ρ*_r_ = 0.45) defines the concentration from which one can observe the ε-Fe_2_O_3_ phase. Finally, the red line represents the value of the laser density necessary to completely achieve the transition towards *α*-Fe_2_O_3_ phase.

The lack of theoretical assignment of the vibrational modes of ε-Fe_2_O_3_ phase allows only a brief discussion and a comparison with vibration bands observed in the literature. Although the vibration modes of ε-Fe_2_O_3_ have not yet been clearly described, the observation of this phase was recently reported on an ancient Jian wares. Indeed, Dejoie et al. [59] showed by TEM/EDX and XRD analyses the presence of this rare metastable polymorph of Fe_2_O_3_ while Raman spectra were collected on large and small crystal clusters. The present Raman spectra, without the maghemite vibration modes, show similarities to those reported by these authors, in particular in the vicinity of the 200–800 cm^−1^ range. However, for a meticulous analysis of the iron oxide phase transitions, an assignment of all vibrational modes of ε-Fe_2_O_3_ phase is now necessary and to our knowledge not accessible yet. Such attribution will help us to understand the relationship between band positions and modifications related to object size, constrained area and chemical composition. A study is in progress to determine all the theoretical Raman active modes for this metastable structure.

## 5. Conclusions

The results presented in this study confirm that the maghemite concentration and the nature of the host matrix play an important role in the stability of the maghemite nanocrystals and can influence the oxidation behavior of iron oxides under either annealing process or *in situ* laser irradiation. We can conclude that γ-Fe_2_O_3_ nanocrystals of 4 nm, dispersed in silica matrix are more stable at low concentrations. Maghemite embedded in silica matrix with low concentration is partially transformed in air to *α*-Fe_2_O_3_ above 1000 °C and the transformation occurs via an indirect mechanism that features ε-Fe_2_O_3_ as an intermediate phase. A structural diagram for the γ-Fe_2_O_3_/SiO_2_ system has been proposed after a detailed analysis of *in situ* laser irradiation investigations using Raman spectroscopy. Samples with *ρ*_r_ ≤  0.45 contain ε-Fe_2_O_3_ phase. Work is in progress to assign the vibrational mode of this metastable structure in order to better follow and to understand the complexity of transitory phases that can appear after different thermal treatments.

## Disclosure statement

No potential conflict of interest was reported by the authors.

## Funding

This work was supported by The Centre de Compétence C’Nano Nord-Ouest, the Collectivités Locales Le Mans Métropole - Département de la Sarthe.

## Supplemental material

The supplemental material for this paper is available online at http://dx.doi.org/10.1080/14686996.2016.1222494

## Supplementary Material

Supplementary.pdf
