# Supplementary material for: Structural behavior of laser-irradiated γ-Fe2O3 nanocrystals dispersed in porous silica matrix : γ-Fe2O3 to α-Fe2O3 phase transition and formation of ε-Fe2O3
Source: Sci Technol Adv Mater. 2016 Sep 29;17(1):597–609. doi: 10.1080/14686996.2016.1222494 (PMC5101921; doi:10.1080/14686996.2016.1222494)
Supplement: Supplementary.pdf [file tsta_a_1222494_sm4619.pdf]

Supplementary Information

To confirm the presence of the ε-Fe_2O_3 phase, we studied the nanocomposite (ρr = 0.15) treated at 1000 °C by Mössbauer spectroscopy at room temperature. The Mössbauer spectrum was recorded using a standard transmission geometry equipped with a conventional constant acceleration spectrometer and a ^{57}Co source diffused into a Rh matrix. The hyperfine parameters were refined by using MOSFIT, a lorentzian linefitting program.[S1]

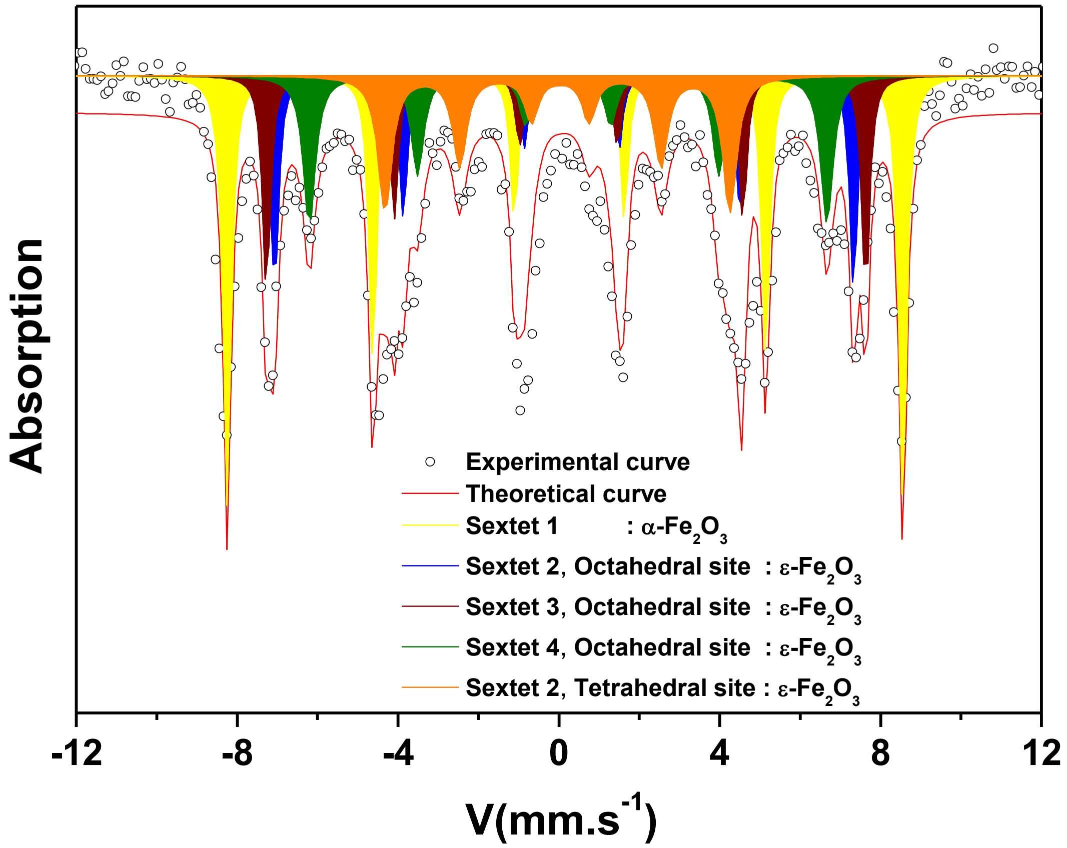

Figure S1 : Mössbauer spectrum obtained at 300 K of the nanocomposite ρr=0.15 treated at 1000 °C recorded at room temperature.

The hyperfine structure can be well described by the presence of five sextets. The refinement parameters with these five components are shown in Table S1. The sextet with the high hyperfine field is assigned unambiguously to hematite. The other four components are characteristic of ε-Fe_2O_3 phase. Their proportions are quite similar, indicating thus the presence of four sites with equal occupation rate, assuming that all of iron atoms in ε-Fe_2O_3 have the same fraction without recoil. The isomer shift values (δ) indicate the existence of three octahedral and one tetrahedral site. Two octahedral sites are characterized by similar values of the hyperfine field, B_{hyp} (≈ 44 T), and different values of the quadrupole displacement 2ε. The third octahedral site has a smaller hyperfine field (≈ 40 T). These values of B_{hyp} are lower than those of the hematite and maghemite (≈50 T).

Componants	%		δ (mm.s^{-1})		2ε (mm.s^{-1})		B_{hyp} (T)	
	**Our study**	Tronc et al.	**Our study**	Tronc et al.	**Our study**	Tronc et al.	**Our study**	Tronc et al.
Sextet 1 (α)	**32 ± 2**	29	***0.36***	*0.37*	**-0.11**	-0.11	***52* ± *1***	*51.5*
Sextet 2 (ε)	**16 ± 2**	17	***0.39***	*0.37*	**-0.19**	-0.19	***44* ± 2**	*45*
Sextet 3 (ε)	**16 ± 2**	17	***0.38***	*0.39*	**-0.06**	-0.06	***46* ± 2**	*45.2*
Sextet 4 (ε)	**17 ± 2**	19	***0.38***	*0.38*	**0**	0	***40* ± 2**	*39.5*
Sextet 5 (ε)	**18 ± 2**	18	***0.21***	*0.21*	**-0.07**	-0.07	***26* ± 2**	*26.2*

Table S1: Refinement parameters obtained with five components of the Mössbauer spectrum, compared to those obtained by Tronc et al. for a similar sample [S2].

Figure S2 : Refinements of the diffraction patterns of three nanocomposites annealed at 1400 °C : ρ_r = 0.04, ρ_r = 0.15 and ρ_r = 0.95.

Sample ρ_r=0.04

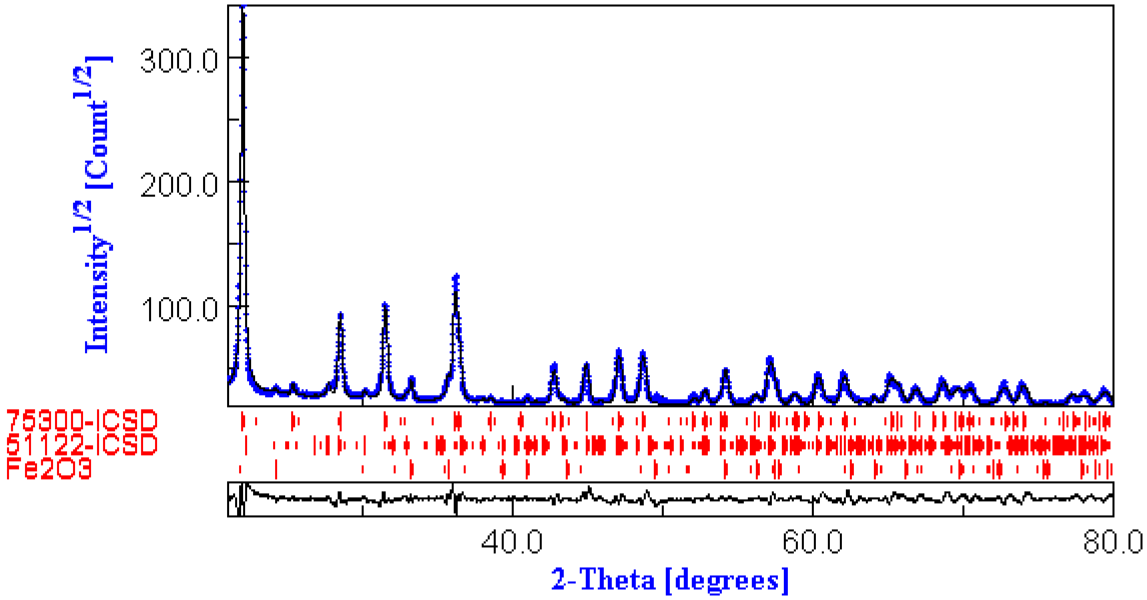

Sample ρ_r=0.15

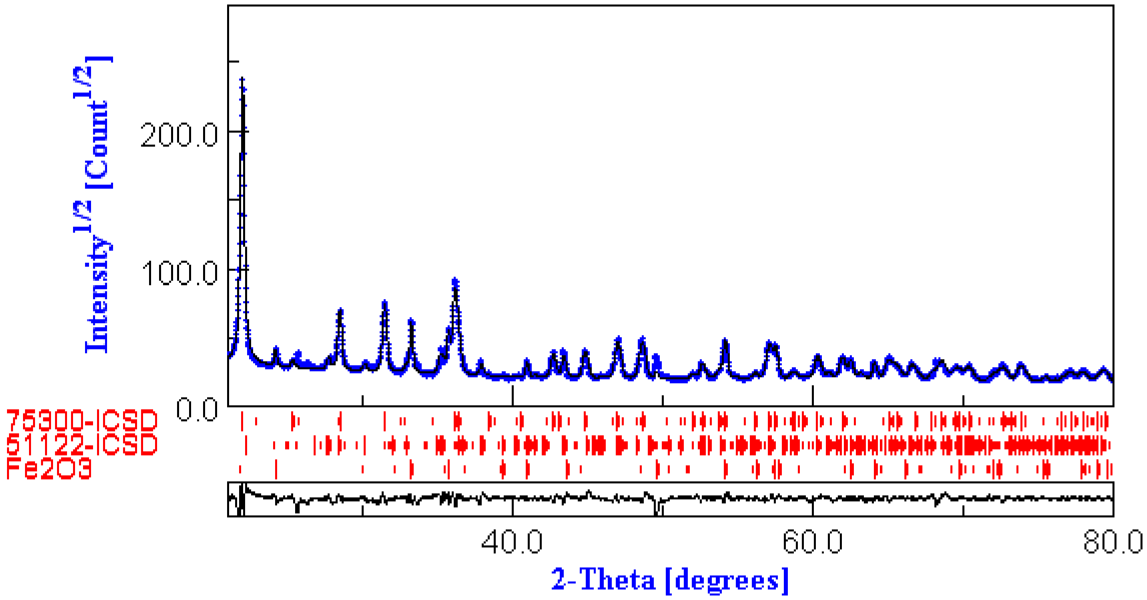

Sample ρ_r=0.95

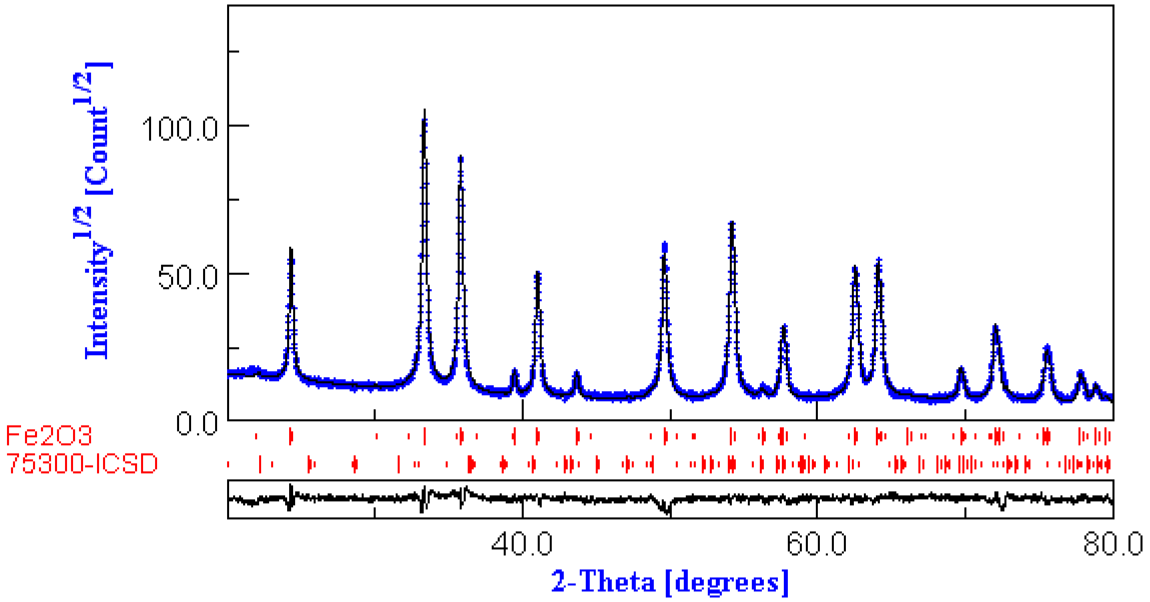

Table S2 : Refined values of lattice parameter of the initial γ-Fe_2O_3/SiO_2 composites annealed at 1400 °C for different mass fraction ρ_r = 0.04, 0.15, 0.95.

	Phase	A,b,c<br>(nm)	<D><br>(nm)	Rms (10^{-4})<br>Microcontrain	%	R_{exp}	R_w	χ_i^2
ρ_r = 0.15	α	a=0.527 (±0.002)<br>b=1.372 (±0.002)	113<br>(±5)	8.8 (±0.5)	8.5	11.86	9.09	1.70
	ε	a=0.510 (±0.002)<br>b=0.878 (±0.002)<br>c=0.946 (±0.002)	55 (±2)	6.61 (±0.5)	4.7			
	SiO_2	a=0.496 (±0.002)<br>b=0.694 (±0.002)	115<br>(±2)	2.72 (±0.5)	86.8			
ρ_r = 0.04	α	a=0.529 (±0.002)<br>b=1.372 (±0.002)	91 (±5)	7.65 (±0.5)	2.3	11.78	9.68	1.48
	ε	a=0.510 (±0.002)<br>b=0.877 (±0.002)<br>c=0.945 (±0.002)	45 (±2)	9.25 (±0.5)	2.6			
	SiO_2	a=0.496 (±0.002)<br>b=0.693 (±0.002)	163<br>(±5)	2.77 (±0.5)	95.1			
ρ_r = 0.95	α	a=0.528 (±0.002)<br>b=1.374 (±0.002)	123<br>(±10)	7.3 (±0.5)	95.3	10.73	7.94	1.82
	SiO_2	a=0.496 (±0.002)<br>b=0.694 (±0.002)	165<br>(±5)	2.71 (±0.5)	4.7			

Complete References

[S1] J. Teillet, F. Varret, Unpublished MOSFIT program.

[S2] E. Tronc, C. Chanéac and J. P Jolivet, Journal of Solid State Chemistry **1998**, 139, 93-104
